# Investigating Antifungal Susceptibility in *Candida* Species With MALDI-TOF MS-Based Assays

**DOI:** 10.3389/fcimb.2019.00019

**Published:** 2019-02-07

**Authors:** Margot Delavy, Andrea R. Dos Santos, Clara M. Heiman, Alix T. Coste

**Affiliations:** Institute of Microbiology, University of Lausanne and University Hospital of Lausanne, Lausanne, Switzerland

**Keywords:** MALDI-TOF MS, *Candida* spp., antifungal resistance, identification, antifungal susceptibility testing, typing

## Abstract

Half of invasive fungal infections lead to death. Amongst pathogenic fungi, the most widespread species belong to the *Candida* genus and vary in their susceptibility to antifungal drugs. The emergence of antifungal resistance has become a major clinical problem. Therefore, the definition of susceptibility patterns is crucial for the survival of patients and the monitoring of resistance epidemiology. Although, most routinely used methods of AntiFungal Susceptibility Testing (AFST) have reached their limits, the rediscovery of Matrix Associated Laser Desorption/Ionization Time of Flight Mass Spectrometry (MALDI-TOF MS) in the field of mycology provides a promising alternative for the study of antifungal resistance. MALDI-TOF MS is already used in mycology for fungal identification, which permits to highlight inherent antifungal resistance. However, the main concern of clinicians is the rise of acquired antifungal resistance and the time needed for their detection. For this purpose, MALDI-TOF MS has been shown to be an accurate tool for AFST, presenting numerous advantages in comparison to commonly used techniques. Finally, MALDI-TOF MS could be used directly to detect resistance mechanisms through typing. Consequently, MALDI-TOF MS offers new perspectives in the context of healthcare associated outbreaks of emerging multi-drug resistant fungi, such as *C. auris*. As a proof of concept, we will illustrate the current and future benefits in using and adapting MALDI-TOF MS-based assays to define the susceptibility pattern of *C. auris*, by species identification, AFST, and typing.

## Introduction

Fungal infections range from harmless superficial infections to invasive illnesses causing generalized sepsis and often leading to death (Brown et al., 2012). Invasive fungal infections are of great concern for clinicians because of the high mortality rate, which reaches up to 50% of patients (Brown et al., 2012). Most of these infections are caused by opportunistic pathogens that take advantage of immunosuppression (HIV/AIDS or cancer patients), even though they are commensal in healthy individuals (Brown et al., 2012). The most prevalent fungal pathogens of invasive infections in these high-risk patients belong to the *Candida* genus (Brown et al., 2012). Moreover, they are the fourth most common cause of nosocomial bloodstream infections. Among *Candida* species (spp.), *C. albicans* is the most prevalent infectious fungus, being responsible for 50–70% of invasive candidiasis (Sanguinetti et al., 2015).

In this review, we will present the current methods used to define the susceptibility patterns of *Candida* spp., by species identification and thus the definition of the inherent susceptibility pattern and by *in vitro* Antifungal Susceptibility Tests (AFST). We will then illustrate how Matrix Associated Laser Desorption/Ionization Time of Flight Mass Spectrometry (MALDI-TOF MS) could be a promising alternative to the routine methods.

## Fungal Infections and Antifungal Resistance

### Treatment of Fungal Infections and Emergence of Antifungal Resistance

Fungal infections can be treated using four main drug classes: azoles, polyenes, pyrimidine analogs, and echinocandins. The first two types of drugs disrupt cell membrane integrity, the third destabilizes nucleic acids whereas the last prevents cell wall biosynthesis (Sanglard, 2016). Precisely, azoles, one of the most broadly used antifungal drug classes, inhibit ergosterol biosynthesis by targeting lanosterol 14-α-sterol demethylase, encoded by the *ERG11* gene in *C. albicans*. This enzyme participates in the conversion of lanosterol to ergosterol, the latter being involved in the cell membrane integrity. By inhibiting the lanosterol 14-α-sterol demethylase, azoles induce the accumulation of a toxic sterol compound, disturbing the fungal cell membrane, leading to growth inhibition (Vandeputte et al., 2012; Sanguinetti et al., 2015). Polyenes bind ergosterol itself, leading to a membrane leakiness, which causes cell death. Finally, Echinocandins bind to β-(1,3)-glucan synthase, inhibiting cell wall synthesis, which also lead to cell death (Sanglard, 2016).

However, as suggested by the high mortality rates, antifungal drugs such as azoles, are not always effective. Indeed, some fungal species are inherently resistant to specific antifungal drugs. Therefore, species identification is crucial and should rapidly be done to select appropriate treatments. Moreover, during long-term treatment, some fungi can acquire new antifungal resistances mechanisms. Even if such resistance is rare among fungi (1–4%), especially in *C. albicans* (1–2% in 2011) (Cleveland et al., 2012; Sanguinetti et al., 2015), they make patient treatment more challenging and need to be rapidly detected. It is particularly important in the case of azole resistance, which has a higher incidence than echinocandin resistance, probably because of the azole fungistatic vs. echinocandin fungicidal properties against *Candida* spp. (Vandeputte et al., 2012).

For a more appropriate patient stewardship, we ideally need to not only rapidly detect the presence of an acquired antifungal resistance but also identify the mechanism involved. This is especially relevant in azole resistance, since it was shown that some resistance mechanisms confer resistance only to some specific azole(s) whereas others confer cross-resistance to all azoles (Sanglard et al., 1995). Therefore, depending on the resistance mechanism identified, the treatment could be more precisely adjusted. Thankfully, during the last 20 years, the four main general mechanisms allowing azole resistance were considerably elucidated (for review, see Vandeputte et al., 2012). The first mechanism consists in the upregulation, via transcription factor (TF) gain-of-function (GOF) mutations, of the expression of ATP-Binding Cassette (ABC) transporters and Major Facilitator Superfamily (MFS) transporters, two types of cell wall transporters responsible for drugs extrusion (Coste et al., 2006; Dunkel et al., 2008). The second mechanism depends on a mutation in Erg11 drug binding site, decreasing the fluconazole affinity and altering the fluconazole antifungal action (Sanglard et al., 1998). The third mechanism involves a GOF mutation in the TF Upc2, leading to the upregulation of *ERG11* expression and the neutralization of the fluconazole action (Flowers et al., 2012). The last mechanism is rarer and relies on yeast metabolism modifications, which inhibits the production of toxic compounds for the fungi (ex: mutation in *ERG3)* (Martel et al., 2010). Echinocandins resistance relies essentially on mutations of *FKS1/FKS2*, coding the β-(1,3)-glucan synthase. Mutations responsible for resistance clustered in two major hot spots. Those mutations lead to reduced affinity of the drug for its target and are associated to pan-echinocandins resistance (Sanglard, 2016).

### Current Routine AFST Methods

Fluconazole resistance is assessed *in vitro* by different Antifungal Susceptibility Tests (AFST). The quality and reproducibility of these analyses are ensured by standardized protocols implemented by the Clinical and Laboratory Standard Institute (USA, CLSI) and the European Committee on Antimicrobial Susceptibility Testing (EUCAST) (Posteraro and Sanguinetti, 2014). CLSI and EUCAST shed light on the clinical success probability for infected patients (Rex et al., 1997; Posteraro and Sanguinetti, 2014).

There exists 3 principal commercial AFST methods (for review see Posteraro and Sanguinetti, 2014): ETEST® (bioMérieux), Sensititre™ YeastOne™ (ThermoFisher Scientific) and the Vitek® 2 Yeast susceptibility system (bioMérieux). The first two are manual minimum inhibitory concentration (MIC) determination methods. The third is an automated system extrapolating a MIC value from 2 to 3 measure points. However, all current routine AFST methods have two main disadvantages. First, they are time-consuming due to the yeast generation-time. Indeed, 24 to 48 h are required to obtain AFST results (Posteraro et al., 2013). Second, results obtained are subjective, particularly for YeastOne™; accurate MIC is difficult to determine and different results can be obtained depending on the experimenter (Marinach et al., 2009; Posteraro and Sanguinetti, 2014). Moreover, the correlations between the clinical breakpoints and the clinical outcomes are not totally reliable (Delarze and Sanglard, 2015). In the case of candidemia, the use of molecular methods like PCR-based methods are not always feasible, even though the mechanisms of antifungal resistance and the gene involved are known (for review, see Vandeputte et al., 2012). In the case of azole resistance, a gene involved in the resistance can be mutated at several locations (Morio et al., 2010; Ferrari et al., 2011; Vandeputte et al., 2012), rendering the elaboration of a PCR-based method laborious or even impossible. Clinical mycology is thus in need of new rapid and objective automated methods to establish the susceptibility level of *Candida* spp. strains, allowing more efficient management of candidemia.

## MALDI-TOF and Antifungal Resistance Detection

MALDI-TOF MS (for review, see Clark et al., 2013) was first used in the late 1990s in clinical microbiology on bacteria (Arnold and Reilly, 1998), since bacterial infections have a higher incidence and bacteria are easier to work with than fungi. MALDI-TOF MS was used, with bacteria, for identification (Arnold and Reilly, 1998), Antimicrobial Susceptibility Testing (AST) (Sparbier et al., 2016) and typing (Arnold and Reilly, 1998; Doern and Butler-Wu, 2016). Since 2001, with the widespread application of MALDI-TOF MS in bacteriology, mycologists attempted to adapt this tool for fungi (Posteraro et al., 2013).

### MALDI-TOF MS for Fungal Identification

The first crucial step in the administration of antifungal therapy is the correct identification of the yeast species. Indeed, as mentioned above all *Candida* spp. strains are not all susceptible to the same antifungal panel. In this context, MALDI-TOF MS is crucial since its first application in clinical microbiology is the rapid and accurate identification of microbial species, especially bacteria (Bader et al., 2011; Posteraro et al., 2013). In the early 2000s, MALDI-TOF MS was rapidly assessed as a useful alternative to classical methods for the identification of not only bacteria but also fungal species (Qian et al., 2008; Marklein et al., 2009; Cassagne et al., 2016). Fungi were more challenging to identify because of their cell wall, which complicates the spectra acquisition. In 2001, protocols were first adapted with *Saccharomyces cerevisiae* (Amiri-Eliasi and Fenselau, 2001). Following this, the idea that MALDI-TOF MS could be automated for fungal identification emerged (Qian et al., 2008). Since 2009, MALDI-TOF MS is routinely used for yeast identification (Marklein et al., 2009; Lee et al., 2011).

To identify a microorganism, the peaks of the experimental spectrum acquired with MALDI-TOF MS are compared to signature peaks from reference spectra, contained in databases. The quality of the identification is dependent on the number of reference spectra available among other factors such as sample growth and preparation, fine settings of the machine, experimenter, etc. (Croxatto et al., 2012). Previous experimental studies demonstrated that MALDI-TOF MS was less arduous than routine identification methods like microscopy, automated blood culture system and biochemical tests to identify *Candida* spp (Marinach et al., 2009). It was shown to be a reliable, fast, and straightforward method compared to conventional ones that are time-consuming and need trained professionals to be interpreted (van Veen et al., 2010; Posteraro et al., 2013). As an illustration, *Candida* spp. can be identified in a day with MALDI-TOF MS whereas other methods can require up to 4 days (Fernandez et al., 2009). Moreover, MALDI-TOF MS analysis was able to differentiate closely related species when conventional biochemical methods were not such as species of the *parapsilosis* complex (*C. parapsilosis, orthopsilosis* and *metapsilosis*) (Bader et al., 2011). Furthermore, MALDI-TOF MS can detect 95.7–100% of common *Candida* species like *C. albicans, C. glabrata, C. dubliniensis*, and *C. tropicalis* (Bader et al., 2011; Bille et al., 2012; Iriart et al., 2012). Accuracy is lower for uncommon species like *C. inconspicua, C. rugosa*, and *C. norvegensis* (73.6–88.9%). However, when databases are sufficiently extensive and regularly updated, MALDI-TOF MS could detect these species, whereas the classical identification method could not (Santos et al., 2011; Posteraro et al., 2013). Indeed, in all the studies mentioned above, MALDI-TOF MS identifications are always at least as good as the classical biochemical identification methods (rapid latex agglutination, ID32 C system (bioMérieux), API 20 C AUX and morphological and carbon source assimilation specificity). The identification of these uncommon species is essential since they are known to have more inherent resistance (Rodloff et al., 2011). Consequently, MALDI-TOF MS offers great benefits in clinical mycology to bring forward inherent resistance through rapid yeast/fungi identification, leading to a first assessment of the antifungal susceptibility level.

### MALDI-TOF MS for AFST

The second crucial step in the administration of appropriate antifungal therapy is the detection of eventual acquired antifungal resistance.

**Figure 1:**
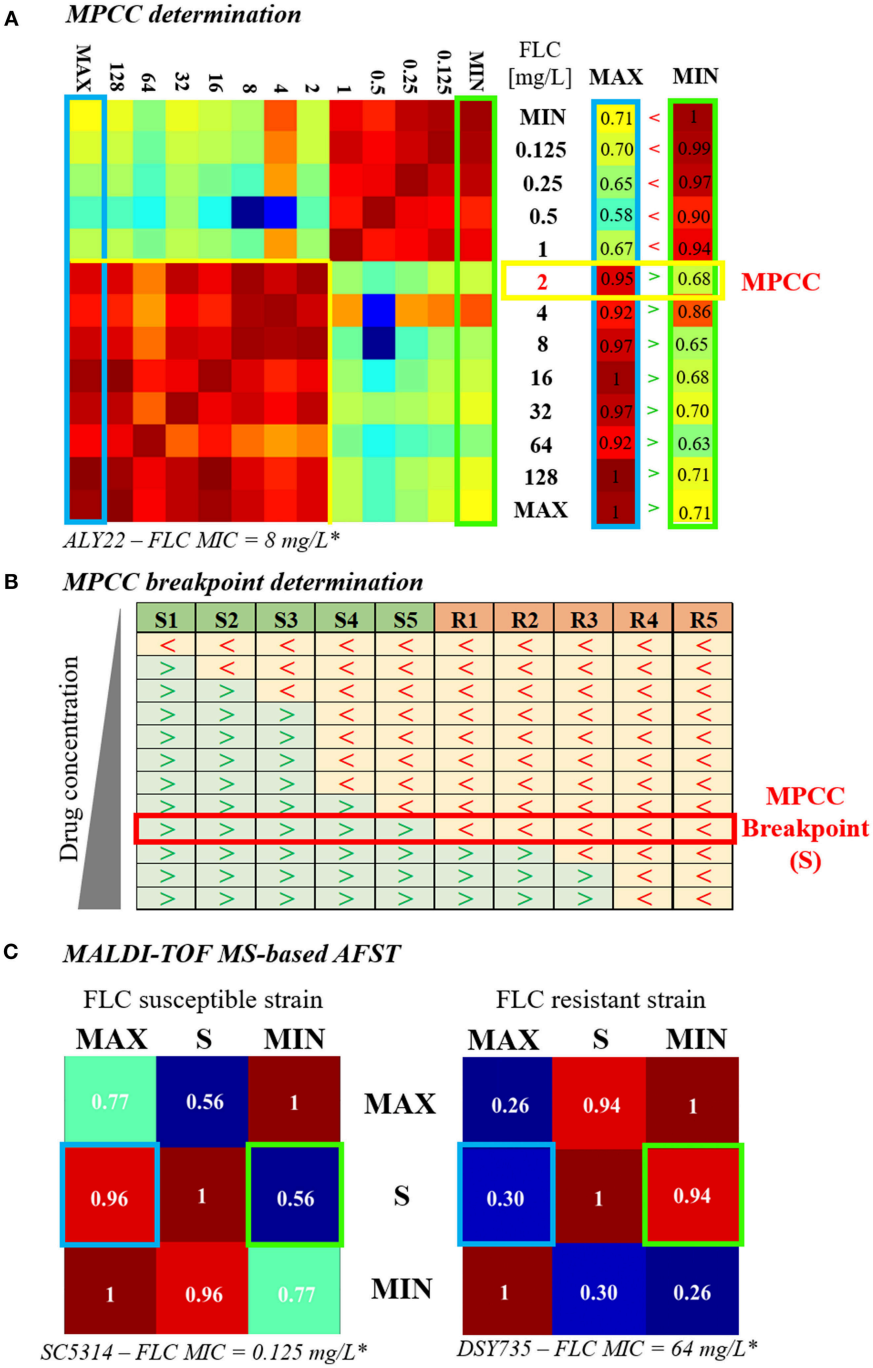
Evolution of the MALDI-TOF MS-based AFST assay. **(A)** MPCC determination. A 13 × 13 CCI matrix (personal data) is generated by comparing the spectra obtained after exposure to increasing concentrations of fluconazole (0 = MIN to 256 μg/mL = MAX), as explained in De Carolis et al. (2012). Red gradient corresponds to strong correlations and blue gradient corresponds to weak ones. The first and the last column of the CCI matrix, namely the comparison of the spectra at all concentrations with the spectra at MAX (blue box) and at MIN (green box), respectively, are isolated. The MPCC (yellow box) corresponds to the fluconazole concentration at which the value of the MAX column is higher (>) than the value of the MIN column, at the same concentration. **(B)** MPCC breakpoint determination. MPCC values of several susceptible (S1 to S5) and resistant (R1 to R5) strains from the same *Candida* spp. are compared. “>” indicates that the spectra at the given concentration is more similar to the spectra at MAX, whereas “ < ” indicates that the spectra at the given concentration is more similar to the spectra at MIN. The MPCC breakpoint concentration (S) correspond to the fluconazole concentration which allow the best discrimination between the resistant and the susceptible strains (red box), as explained in De Carolis et al. (2012). **(C)** MALDI-TOF MS-based assay. A 3 × 3 CCI matrix is generated by comparing the spectra at MAX, S, and MIN, as presented in Vella et al. (2013). The results of the correlation between S and MAX (blue box) and S and MIN (green box) are compared. The strain is assessed as susceptible if the result of the correlation between S an MAX is higher than the result of the correlation between S and MIN (left matrix, personal data), whereas the strain is assessed as resistant if the result of the correlation between S an MAX is lower than the result of the correlation between S and MIN (right matrix, personal data).^*^Lohberger et al. (2014), FLC = fluconazole.

In 2009, Marinach et al. developed a first MALDI-TOF MS-based assay to discriminate fluconazole-resistant *C. albicans* strains from susceptible ones. Their method is based on the determination of the minimal profile change concentration (MPCC) for a given strain, a new alternative endpoint to the classical MIC. It was shown than MPCC and MIC are correlated, presenting a discrepancy of maximum 2 dilutions. MPCC is defined as the minimal drug concentration needed to detect changes in MALDI-TOF MS spectra. MPCC corresponds to the minimal concentration at which the result of the cross-correlation with the spectra at the maximal concentration (128 μg/mL for *C. albicans* and fluconazole) is higher than the result of the cross-correlation with the spectra at the null concentration (0 μg/mL) (Figure 1A, right panel; Marinach et al., 2009). The MPCC determination was then made more objective and quantitative by De Carolis et al. (2012), using a composite correlation index (CCI) matrix obtained with the MALDI Biotyper® software. The CCI matrix provides scores of composite correlation between all the MALDI-TOF MS spectra allowing a quantitative determination of the MPCC, which is also better visualized (Figure 1A; De Carolis et al., 2012). MPCC breakpoints were then defined as the minimal antifungal drug concentration at which all spectra from the known resistant strains are more similar to the spectra at the null concentration, whereas all spectra from the known susceptible strains are more similar to the spectra at the maximal concentration (Figure 1B). MPCC breakpoints are species and drug-dependent, like the EUCAST and CLSI breakpoints, and allow to interpret MALDI-TOF MS AFST results (De Carolis et al., 2012). This new MALDI-TOF MS-based assay was improved by Vella et al. (2013). They decreased the exposure time to the antifungal drug from 15 h (De Carolis et al., 2012) to 3 h and simplified the CCI matrix to a 3 × 3 matrix (Figure 1C), including spectra at maximal (MAX), null (MIN), and MPCC breakpoint (S) drug concentrations. A yeast strain is diagnosed as resistant if the CCI between S and MIN is higher than the CCI between S and MAX (Vella et al., 2013). Finally, Vella et al. tested their method on different drugs (caspofungin, anidulafungin, and fluconazole) and different *Candida* spp (*C. albicans* and *C. glabrata)*. Even if they obtained partial success for the assessment of the anidulafungin susceptibility (from 25 to 100% of accuracy, depending on the mutated gene), they achieve a highly accurate determination of the fluconazole and caspofungin susceptibilities (more than 90% success; Vella et al., 2013, 2017).

Alternatively, a new MALDI-TOF MS-based susceptibility assays was shown to accurately discriminate resistant from susceptible strains in 6 h (Vatanshenassan et al., 2018). Indeed, MALDI Biotyper antibiotic susceptibility test rapid assay (MBT-ASTRA) was initially developed with bacteria and was able to assess a susceptibility level depending on the cellular growth, quantified by the peak intensities of the MALDI-TOF MS spectra. Hence, a susceptible strain would display lower peaks intensities, representing the lack of growth whereas resistant strains would display higher peaks intensities as compared to an internal standard (Sparbier et al., 2016). MBT-ASTRA was then adapted to assess caspofungin susceptibility in *C. albicans* and *C. glabrata*, with an accuracy ranging from 80 to 100% (Vatanshenassan et al., 2018).

Thus, the reliability, accuracy and objectivity of the results interpretation of MALDI-TOF MS-based assays offer advantages over other existing AFST methods. Moreover, the time saved by both MALDI-TOF MS-based assays presented, with results obtained in 3 to 15 h, in comparison to 48 h for other methods (Posteraro and Sanguinetti, 2014), is crucial for clinicians. However, to be optimal, both tools need further development to be able to detect resistance to several antifungal drugs at a time for a broader range of yeast species, in a simplified routine use. To this end, kits and software should be combined and developed to be more accessible and allow autonomous analyses of results. Another lead might be the coupling of total laboratory automation with MALDI-TOF MS in the classic workflow of the diagnostic routine labs (Theparee et al., 2018). Nevertheless, MALDI-TOF-MS is on the edge of becoming an indispensable tool in the field of AFST.

### MALDI-TOF MS for Typing

As we explained above, MALDI-TOF MS is a promising tool for the detection of acquired antifungal resistance. However, the previously presented MALDI-TOF MS-based AFST assays require incubation with antifungal, which is not easy in routine. Ideally, the susceptibility of a *Candida* spp. strain should be determined directly by spectra sub-typing, meaning detecting specific peaks associated with resistance.

Therefore, the first step would be, as it was already done for some bacteria (Manukumar and Umesha, 2017), to detect specific peaks associated with the resistance or the susceptibility to an antifungal drug (Posteraro et al., 2013). This aim is promising since typing with MALDI-TOF MS was shown to be able to cluster *C. glabrata* isolates according to their fluconazole susceptibility profile (Dhieb et al., 2015) but no resistance nor susceptibility peaks has yet been identified. In a further step, identification of the resistance mechanism will further increase the efficacy of the antifungal stewardship. Indeed, in the case of diploid fungi such as *C. albicans*, a single copy GOF mutation is sometimes phenotypically undetectable (Coste et al., 2006). However, resistance can arise rapidly if the yeast becomes homozygous for the resistance mutation or by increasing the copy number of the gene carrying the GOF mutation (Coste et al., 2006; Selmecki et al., 2010). Thus, we could detect resistance mechanisms even before resistance is phenotypically expressed hence improving antifungal stewardship. Moreover, typing with MALDI-TOF MS could be used to track the geographical origin of a strain, which is of great epidemiological value. To date, to the authors' knowledge, one single study in this field was reported for fungi (Dhieb et al., 2015). However, this will be especially relevant in the case of the threat generated by potential outbreak-causing emerging fungi such as *C. auris*.

## MALDI-TOF, a Crucial Tool to Manage the Emerging Pathogen *Candida auris*

*C. auris* was first identified in Japan in 2009 (Satoh et al., 2009). To date, four clades of *C. auris* have been identified (Sarma and Upadhyay, 2017), impacting several countries in the Americas, Africa, Asia, and Europe (Jeffery-Smith et al., 2018). It quickly became a major clinical threat because of its ability to spread from patient to patient in hospital settings and its high level of antifungal resistance, classifying it as a multidrug-resistant species (Lee et al., 2011; Schelenz et al., 2016; Araúz et al., 2017). Moreover, *C. auris* infections are even more complex to treat as they are often misdiagnosed (Jeffery-Smith et al., 2018). Therefore, it is essential to rapidly identify and determine antifungal susceptibility profile of *C. auris* isolates to optimize patient care and implement appropriate hospital hygiene measures.

**Figure 2:**
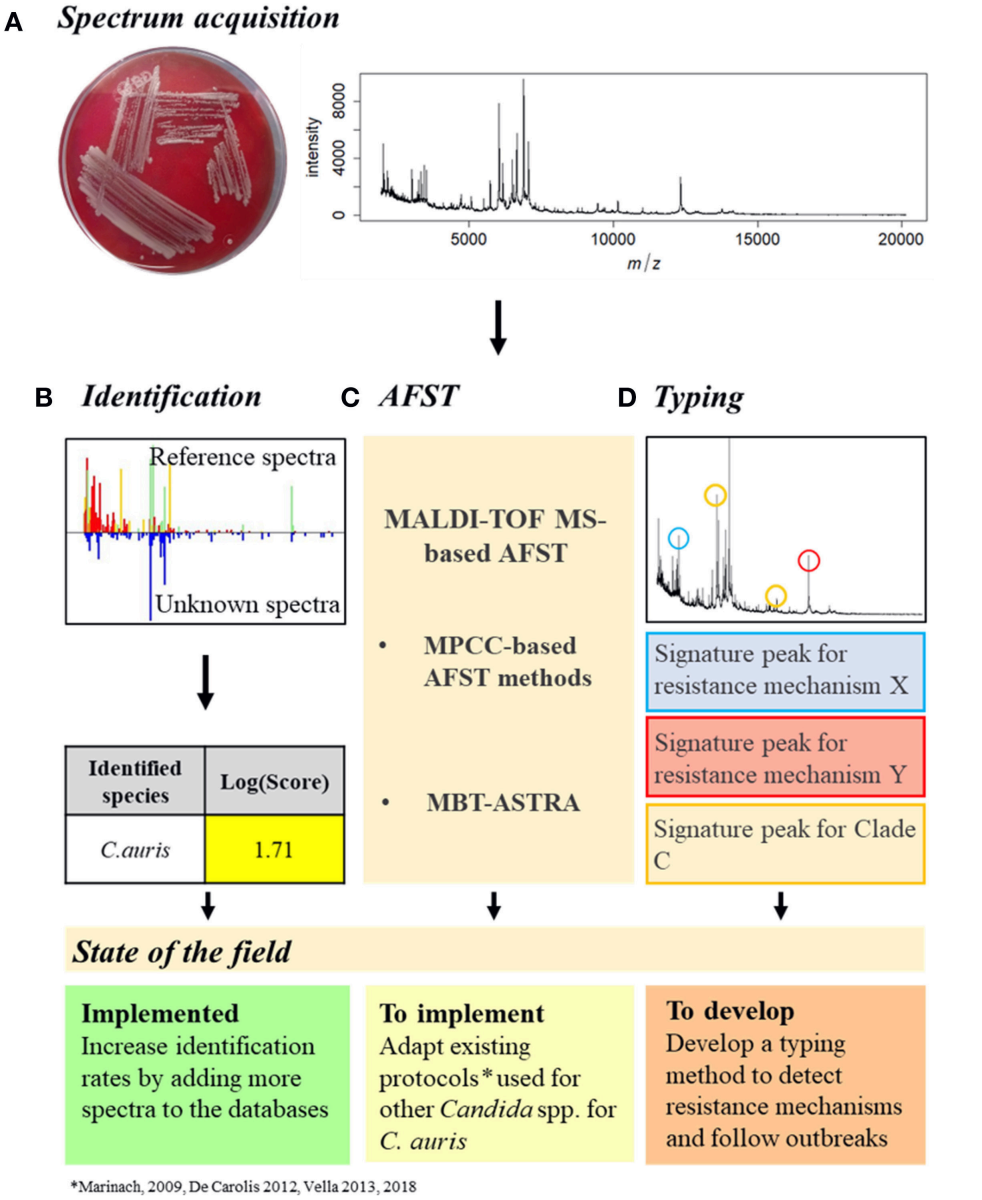
Proposition of *C. auris* outbreak management with MALDI-TOF MS. **(A)** Spectrum acquisition. A *C. auris* colony (here grown on Columbia medium) is analyzed by MALDI-TOF MS to obtain a spectrum. **(B)** Identification. The spectrum obtained is compared to the reference spectra of the databases and an identification Log(Score) is calculated. For fungal species, a proposed identification is accepted if the Log(Score) is equal or higher than 1.7. *C. auris* identification with MALDI-TOF MS is already implemented but needs improvements. **(C)** AFST. Spectra obtained at different antifungal concentrations need to be compared to determine the MPCC. From there, once the EUCAST and/or CLSI breakpoints have been defined, the MPCC breakpoint for *C. auris* and an antifungal drug can be determined, allowing the AFST implementation. AFST with MALDI-TOF MS need to be adapted from existing protocols (Marinach et al., 2009; De Carolis et al., 2012; Vella et al., 2013, 2017) to be used on *C. auris*. **(D)** Typing. Spectra of different *C. auris* strains originated from different clades and with different resistance mechanisms could be compared to identify signature peaks specific to different resistance mechanisms or different clades. *C. auris* typing with MALDI-TOF MS needs to be developed.

To manage a *C. auris* outbreak, the first step is the identification of the pathogen (Figures 2A,B). MALDI-TOF MS has been proven to be the most accurate available method to identify *C. auris*, surpassing all the other identification methods (Jeffery-Smith et al., 2018). However, MALDI-TOF MS-based identification still does not allow a sufficient identification rate, leading to misdiagnosis (Chowdhary et al., 2017). This is due to the lack of *C. auris* reference spectra in the databases (Mizusawa et al., 2017). Nevertheless, this issue could be easily resolved by increasing the number of reference spectra available (Jeffery-Smith et al., 2018). Accordingly, Bruker recently released an updated library, containing a total of 9 *C. auris* spectra, but only in the Research Only Database (RUO).

Once a *C. auris* infection is confirmed, AFST need to be regularly performed to detect the appearance of antifungal resistance (Figure 2C). However, as no official clinical breakpoint has yet been assessed for *C. auris*, either by EUCAST or CLSI (CLSI, 2017; EUCAST, 2018), current AFST methods are unable to accurately discriminate resistant from susceptible strains (Jeffery-Smith et al., 2018). Therefore, since MALDI-TOF MS-based AFST protocols have already been developed for several drugs and *Candida* spp (Marinach et al., 2009; De Carolis et al., 2012; Vella et al., 2013, 2017), once *C. auris* breakpoints have been defined, MALDI-TOF MS-based protocols could be adapted. Finally, MALDI-TOF MS-based typing could allow identification of signature peaks associated with the different clades or even strains, providing an easy tool to follow an outbreak by tracking its origin and its evolution, linked to a susceptibility profile, allowing a better antifungal management of the outbreak (Figure 2D).

To conclude, fungal infections are challenging, especially with the complications brought up by antifungal resistance. However, MALDI-TOF MS has already revolutionized the diagnosis of fungal infections and future developments would bring new methods to assess the susceptibility patterns of fungal infections, leading to a new era in clinical mycology.

## Author Contributions

MD, AD, and CH conducted a literature review exercise as part of their Master's degree studies at the University of Lausanne under the supervision of AC.

### Conflict of Interest Statement

The authors declare that the research was conducted in the absence of any commercial or financial relationships that could be construed as a potential conflict of interest.

## References

[B1] Amiri-Eliasi B. Fenselau C. (2001). Characterization of protein biomarkers desorbed by MALDI from whole fungal cells. Anal. Chem. 73, 5228–5231. 10.1021/ac010651t 11721923

[B2] Araúz A. B. Caceres D. H. Santiago E. Armstrong P. Arosemena S. Ramos C.. (2017). Isolation of Candida Auris from 9 patients in central america: importance of accurate diagnosis and susceptibility testing. Mycoses 61, 44–47. 10.1111/myc.12709 28945325

[B3] Arnold R. J. Reilly J. P. (1998). Fingerprint matching of *E. Coli* strains with matrix-assisted laser desorption/ionization time-of-flight mass spectrometry of whole cells using a modified correlation approach. Rapid Commun. Mass Spectrom. RCM 12, 630–636. 10.1002/(SICI)1097-0231(19980529)12:10<630::AID-RCM206>3.0.CO;2-0 9621446

[B4] Bader O. Weig M. Taverne-Ghadwal L. Lugert R. Groß U. Kuhns M. (2011). Improved clinical laboratory identification of human pathogenic yeasts by matrix-assisted laser desorption ionization time-of-flight mass spectrometry. Clin. Microbiol. Infect. 17, 1359–1365. 10.1111/j.1469-0691.2010.03398.x 20946411

[B5] Bille E. Dauphin B. Leto J. Bougnoux M.-E. Beretti J.-L. Lotz A. Suarez S.. (2012). MALDI-TOF MS andromas strategy for the routine identification of bacteria, mycobacteria, yeasts, aspergillus spp. and positive blood cultures. Clin. Microbiol. Infect. 18, 1117–1125. 10.1111/j.1469-0691.2011.03688.x 22044600

[B6] Brown G. D. Denning D. W. Gow N. A. R. Levitz S. M. Netea M. G. White T. C. (2012). Hidden killers: human fungal infections. Sci. Trans. Med. 4:165rv13. 10.1126/scitranslmed.3004404 23253612

[B7] Cassagne C. Normand A.-C. Stéphane Ranque C. L. Piarroux R. (2016). Performance of MALDI-TOF MS platforms for fungal identification. Mycoses 59, 678–690. 10.1111/myc.12506 27061755

[B8] Chowdhary A. Sharma C. Meis J. F. (2017). *Candida Auris*: a rapidly emerging cause of hospital-acquired multidrug-resistant fungal infections globally. PLOS Pathogens 13:e100. 10.1371/journal.ppat.1006290 28542486 PMC5436850

[B9] Clark A. E. Kaleta E. J. Arora A. Wolk D. M. (2013). Matrix-assisted laser desorption ionization–time of flight mass spectrometry: a fundamental shift in the routine practice of clinical microbiology. Clin. Microbiol. Rev. 26, 547–603. 10.1128/CMR.00072-12 23824373 PMC3719498

[B10] Cleveland A. A. Farley M. M. Harrison L. H. Stein B. Hollick R. Lockhart S. R.. (2012). Changes in incidence and antifungal drug resistance in candidemia: results from population-based laboratory surveillance in atlanta and baltimore, 2008-2011. Clin. Infect. Dis. 55, 1352–1361. 10.1093/cid/cis697 22893576 PMC4698872

[B11] CLSI (2017). Performance Standards for Antifungal Susceptibility Testing of Yeasts. CLSI Supplement M60, 1st Edn. Wayne, PA: Clinical and Laboratory Standard Institute.

[B12] Coste A. Turner V. Ischer F. Morschhäuser J. Forche A. Selmecki A.. (2006). A mutation in Tac1p, a transcription factor regulating CDR1 and CDR2, is coupled with loss of heterozygosity at chromosome 5 to mediate antifungal resistance in *Candida Albicans*. Genetics 172, 2139–2156. 10.1534/genetics.105.054767 16452151 PMC1456413

[B13] Croxatto A. Prod'hom G. Greub G. (2012). Applications of MALDI-TOF mass spectrometry in clinical diagnostic microbiology. FEMS Microbiol. Rev. 36, 380–407. 10.1111/j.1574-6976.2011.00298.x 22092265

[B14] De Carolis E. Vella A. Florio A. R. Posteraro P. Perlin D. S. Sanguinetti M.. (2012). Use of matrix-assisted laser desorption ionization-time of flight mass spectrometry for caspofungin susceptibility testing of candida and aspergillus species. J. Clin. Microbiol. 50, 2479–2483. 10.1128/JCM.00224-12 22535984 PMC3405623

[B15] Delarze E. Sanglard D. (2015). Defining the frontiers between antifungal resistance, tolerance and the concept of persistence. Drug Resist. Updates 23 (Suppl. C), 12–19. 10.1016/j.drup.2015.10.001 26690338

[B16] Dhieb C. Normand A. C. Al-Yasiri M. Chaker E. El Euch D. Vranckx K.. (2015). MALDI-TOF typing highlights geographical and fluconazole resistance clusters in candida glabrata. Med. Mycol. 53, 462–469. 10.1093/mmy/myv013 25841053

[B17] Doern C. D. Butler-Wu S. M. (2016). Emerging and future applications of matrix-assisted laser desorption ionization time-of-flight (MALDI-TOF) mass spectrometry in the clinical microbiology laboratory: a report of the association for molecular pathology. J. Mol. Diagn. 18, 789–802. 10.1016/j.jmoldx.2016.07.007 27770851

[B18] Dunkel N. Liu T. T. Barker K. S. Homayouni R. Morschhäuser J. David Rogers P. (2008). Gain-of-function mutation in the transcription factor Upc2p causes upregulation of ergosterol biosynthesis genes and increased fluconazole resistance in a clinical candida albicans isolate. Eukaryotic Cell 7, 1180–1190. 10.1128/EC.00103-08 18487346 PMC2446669

[B19] EUCAST (2018). EUCAST: About “Clinical Breakpoints”. EUCAST- European Society of Clinical Microbiology and Infectious Diseases. 2018. Available online at: http://www.eucast.org/clinical_breakpoints/about_clinical_breakpoints/

[B20] Fernandez J. Erstad B. L. Petty W. Nix D. E. (2009). Time to positive culture and identification for candida blood stream infections. Diagn. Microbiol. Infect. Dis. 64, 402–407. 10.1016/j.diagmicrobio.2009.04.002 19446982

[B21] Ferrari S. Sanguinetti M. De Bernardis F. Torelli R. Posteraro B. Vandeputte P.. (2011). Loss of mitochondrial functions associated with azole resistance in candida glabrata results in enhanced virulence in mice? Antimicrob. Agents Chemother. 55, 1852–1860. 10.1128/AAC.01271-10 21321146 PMC3088236

[B22] Flowers S. A. Barker K. S. Berkow E. L. Toner G. Chadwick S. G. Gygax S. E.. (2012). Gain-of-function mutations in UPC2 are a frequent cause of ERG11 upregulation in azole-resistant clinical isolates of *Candida Albicans*. Eukaryotic Cell 11, 1289–1299. 10.1128/EC.00215-12 22923048 PMC3485914

[B23] Iriart X. Lavergne R.-A. Fillaux J. Valentin A. Magnaval J.-F. Berry A.. (2012). Routine identification of medical fungi by the new vitek MS matrix-assisted laser desorption ionization–time of flight system with a new time-effective strategy. J. Clin. Microbiol. 50, 2107–2110. 10.1128/JCM.06713-11 22495559 PMC3372123

[B24] Jeffery-Smith A. Taori S. K. Schelenz S. Jeffery K. Johnson E. M. Borman A.. (2018). *Candida Auris*: a review of the literature. Clin. Microbiol. Rev. 31:e00029–17. 10.1128/CMR.00029-17 29142078 PMC5740969

[B25] Lee W. G. Shin J. H. Uh Y. Kang M. G. Kim S. H. Park K. H.. (2011). First three reported cases of nosocomial fungemia caused by *Candida Auris*. J. Clin. Microbiol. 49, 3139–3142. 10.1128/JCM.00319-11 21715586 PMC3165631

[B26] Lohberger A. Coste A. T. Sanglard D. (2014). Distinct roles of *Candida Albicans* drug resistance transcription factors TAC1, MRR1, and UPC2 in virulence. Eukaryotic Cell 13, 127–142. 10.1128/EC.00245-13 24243794 PMC3910953

[B27] Manukumar H. M. Umesha S. (2017). MALDI-TOF-MS based identification and molecular characterization of food associated methicillin-resistant *Staphylococcus Aureus*. Sci. Rep. 7:1. 10.1038/s41598-017-11597-z 28900246 PMC5595867

[B28] Marinach C. Alanio A. Palous M. Kwasek S. Fekkar A. Brossas J.-Y.. (2009). MALDI-TOF MS-based drug susceptibility testing of pathogens: the example of *Candida Albicans* and fluconazole. Proteomics 9, 4627–4631. 10.1002/pmic.200900152 19750514

[B29] Marklein G. Josten M. Klanke U. Müller E. R. Horré T. Maier T. Wenzel. (2009). Matrix-assisted laser desorption ionization-time of flight mass spectrometry for fast and reliable identification of clinical yeast isolates. J. Clin. Microbiol. 47, 2912–2917. 10.1128/JCM.00389-09 19571014 PMC2738125

[B30] Martel C. M. Parker J. E. Bader O. Weig M. Gross U. Warrilow A. G. S.. (2010). Identification and characterization of four azole-resistant Erg3 mutants of *Candida Albicans*. Antimicrob. Agents Chemother. 54, 4527–4533. 10.1128/AAC.00348-10 20733039 PMC2976150

[B31] Mizusawa M. Miller H. Green R. Lee R. Durante M. Perkins R.. (2017). Can multidrug-resistant *Candida Auris* be reliably identified in clinical microbiology laboratories? Edited by Warnock David W. J. Clin. Microbiol. 55, 638–640. 10.1128/JCM.02202-16 27881617 PMC5277535

[B32] Morio F. Loge C. Besse B. Hennequin C. Le Pape P. (2010). Screening for amino acid substitutions in the *Candida Albicans* Erg11 protein of azole-susceptible and azole-resistant clinical isolates: new substitutions and a review of the literature. Diagn. Microbiol. Infect. Dis. 66, 373–384. 10.1016/j.diagmicrobio.2009.11.006 20226328

[B33] Posteraro B. De Carolis E. Vella A. Sanguinetti M. (2013). MALDI-TOF mass spectrometry in the clinical mycology laboratory: identification of fungi and beyond. Expert Rev. Proteomics 10, 151–164. 10.1586/epr.13.8 23573782

[B34] Posteraro B. Sanguinetti M. (2014). The future of fungal susceptibility testing. Future Microbiol. 9, 947–967. 10.2217/fmb.14.55 25302953

[B35] Qian J. Cutler J. E. Cole R. B. Cai Y. (2008). MALDI-TOF mass signatures for differentiation of yeast species, strain grouping and monitoring of morphogenesis markers. Anal. Bioanal. Chem. 392, 439–449. 10.1007/s00216-008-2288-1 18690424

[B36] Rex J. H. Pfaller M. A. Galgiani J. N. Bartlett M. S. Espinel-Ingroff A. Ghannoum M. A.. (1997). Development of interpretive breakpoints for antifungal susceptibility testing: conceptual framework and analysis of *in vitro*-*in vivo* correlation data for fluconazole, itraconazole, and candida infections. Subcommittee on antifungal susceptibility testing of the National Committee for clinical laboratory standards. Clin. Infect. Dis. 24, 235–247. 10.1093/clinids/24.2.235 9114154

[B37] Rodloff A. C. Koch D. Schaumann R. (2011). Epidemiology and antifungal resistance in invasive candidiasis. Eur. J. Med. Res. 16, 187–195. 10.1186/2047-783X-16-4-187 21486733 PMC3352075

[B38] Sanglard D. (2016). Emerging threats in antifungal-resistant fungal pathogens. Front. Med. 3:11. 10.3389/fmed.2016.00011 27014694 PMC4791369

[B39] Sanglard D. Ischer F. Koymans L. Bille J. (1998). Amino acid substitutions in the cytochrome P-450 lanosterol 14alpha-demethylase (CYP51A1) from azole-resistant *Candida Albicans* clinical isolates contribute to resistance to azole antifungal agents. Antimicrob. Agents Chemother. 42, 241–253. 9527767 10.1128/aac.42.2.241 PMC105395

[B40] Sanglard D. Kuchler K. Ischer F. Pagani J. L. Monod M. Bille J. (1995). Mechanisms of resistance to azole antifungal agents in *Candida Albicans* isolates from AIDS patients involve specific multidrug transporters. Antimicrob. Agents Chemother. 39, 2378–2386. 10.1128/AAC.39.11.2378 8585712 PMC162951

[B41] Sanguinetti M. Posteraro B. Lass-Flörl C. (2015). Antifungal drug resistance among Candida species: mechanisms and clinical impact. Mycoses 58(Suppl 2), 2–13. 10.1111/myc.12330 26033251

[B42] Santos C. Lima N. Sampaio P. Pais C. (2011). Matrix-assisted laser desorption/ionization time-of-flight intact cell mass spectrometry to detect emerging pathogenic *Candida* species. Diagn. Microbiol. Infect. Dis. 71, 304–308. 10.1016/j.diagmicrobio.2011.07.002 21855250

[B43] Sarma S. Upadhyay S. (2017). Current perspective on emergence, diagnosis and drug resistance in *Candida Auris*. Infect. Drug Resist. 10, 155–165. 10.2147/IDR.S116229 28652784 PMC5476417

[B44] Satoh K. Makimura K. Hasumi Y. Nishiyama Y. Uchida K. Yamaguchi H. (2009). *Candida Auris* Sp. Nov., a novel ascomycetous yeast isolated from the external ear canal of an inpatient in a Japanese Hospital. Microbiol. Immunol. 53, 41–44. 10.1111/j.1348-0421.2008.00083.x 19161556

[B45] Schelenz S. Hagen F. Rhodes J. L. Abdolrasouli A. Chowdhary A. Hall A.. (2016). First hospital outbreak of the globally emerging *Candida Auris* in a european hospital. Antimicrob. Resist. Infect. Control 5:35. 10.1186/s13756-016-0132-5 27777756 PMC5069812

[B46] Selmecki A. Forche A. Berman J. (2010). Genomic plasticity of the human fungal pathogen *Candida albicans*. Eukaryotic Cell 9, 991–1008. 10.1128/EC.00060-10 20495058 PMC2901674

[B47] Sparbier K. Schubert S. Kostrzewa M. (2016). MBT-ASTRA: a suitable tool for fast antibiotic susceptibility testing? Methods 104 (Suppl. C), 48–54. 10.1016/j.ymeth.2016.01.008 26804565

[B48] Theparee T. Das S. Thomson R. B. (2018). Total laboratory automation and matrix-assisted laser desorption ionization–time of flight mass spectrometry improve turnaround times in the clinical microbiology laboratory: a retrospective analysis. J. Clin. Microbiol. 56:e01242–17. 10.1128/JCM.01242-17 29118171 PMC5744220

[B49] van Veen S. Q. van Claas E. C. J. Kuijper J. (2010). High-throughput identification of bacteria and yeast by matrix-assisted laser desorption ionization-time of flight mass spectrometry in conventional medical microbiology laboratories. J. Clin. Microbiol. 48, 900–907. 10.1128/JCM.02071-09 20053859 PMC2832429

[B50] Vandeputte P. Ferrari S. Coste A. T. (2012). Antifungal resistance and new strategies to control fungal infections. Int. J. Microbiol. 2012:71. 10.1155/2012/713687 22187560 PMC3236459

[B51] Vatanshenassan M. Boekhout T. Lass-Flörl C. Lackner M. Schubert S. Kostrzewa M. (2018). MBT ASTRA: proof-of-concept for a rapid MALDI-TOF MS based method to detect caspofungin resistance in *Candida Albicans* and *Candida Glabrata*. J. Clin. Microbiol. 420:18 10.1128/JCM.00420-18 PMC6113492 30021820

[B52] Vella A. De Carolis E. Mello E. Perlin D. S. Sanglard D. Sanguinetti M.. (2017). Potential use of MALDI-ToF mass spectrometry for rapid detection of antifungal resistance in the human pathogen *Candida Glabrata*. Sci. Rep. 7:9099. 10.1038/s41598-017-09329-4 28831086 PMC5567316

[B53] Vella A. De Carolis E. Vaccaro L. Posteraro P. Perlin D. S. Kostrzewa M.. (2013). Rapid antifungal susceptibility testing by matrix-assisted laser desorption ionization-time of flight mass spectrometry analysis. J. Clin. Microbiol. 51, 2964–2969. 10.1128/JCM.00903-13 23824764 PMC3754633

